# Neural Crest-Derived Chondrocytes Isolation for Tissue Engineering in Regenerative Medicine

**DOI:** 10.3390/cells9040962

**Published:** 2020-04-14

**Authors:** Monica Salamone, Salvatrice Rigogliuso, Aldo Nicosia, Marcello Tagliavia, Simona Campora, Paolo Cinà, Carmelo Bruno, Giulio Ghersi

**Affiliations:** 1 Abiel s.r.l, c/o University of Palermo, Viale delle Scienze, Ed. 16, 90128 Palermo, Italy; m.salamone@abielbiotech.com (M.S.); silvia.rigogliuso@gmail.com (S.R.); simona.campora@unipa.it (S.C.); paolo.cina86@gmail.com (P.C.); brunocarmelo@hotmail.com (C.B.); 2 Institute for Biomedical Research and Innovation-National Research Council (IRIB-CNR), Via Ugo La Malfa 153, 90146 Palermo, Italy; aldo.nicosia@cnr.it (A.N.); marcello.tagliavia@cnr.it (M.T.); 3 Department of Biological, Chemical and Pharmaceutical Sciences and Technologies [STEBICEF], University of Palermo, Viale delle Scienze, Ed. 16, 90128 Palermo, Italy

**Keywords:** nasal chondrocytes, tissue dissociation, collagenases, gene expression profiles, cell transplantation

## Abstract

Chondrocyte transplantation has been successfully tested and proposed as a clinical procedure aiming to repair articular cartilage defects. However, the isolation of chondrocytes and the optimization of the enzymatic digestion process, as well as their successful in vitro expansion, remain the main challenges in cartilage tissue engineering. In order to address these issues, we investigated the performance of recombinant collagenases in tissue dissociation assays with the aim of isolating chondrocytes from bovine nasal cartilage in order to establish the optimal enzyme blend to ensure the best outcomes of the overall procedure. We show, for the first time, that collagenase H activity alone is required for effective cartilage digestion, resulting in an improvement in the yield of viable cells. The extracted chondrocytes proved able to grow and activate differentiation/dedifferentiation programs, as assessed by morphological and gene expression analyses.

## 1. Introduction

Organ transplant therapy is currently hampered by the limited availability of compatible donors, and often by the onset of severe immune complications. In some instances, these limitations can be—at least partially—overcome by approaches which rely on regenerative medicine, including gene and cell therapies [1]. 

Chondrocytes have been shown to be among the most versatile cell types due to their renewal/differentiation ability, which makes them suitable for various applications [2]. In this context, autologous chondrocyte implantation (ACT) is considered the gold standard for cartilage repair [3]; moreover, recent preclinical studies have provided promising evidence for the effectiveness of cell-based regeneration strategies [4,5]. 

More recently proposed approaches for cartilage regeneration include matrix-associated autologous chondrocyte implantation (MACI) and autologous matrix-induced chondrogenesis (AMIC) [6]. 

Adult Nasal Chondrocytes (NC) are considered particularly worthy of attention due to their unique features [7]. They derive from the neural crest, and have been recently shown to be able to respond and adapt to heterotopic transplantation sites. In particular, chondrocytes from the nasal septum have shown improved reproducibility in generating hyaline-like cartilage tissues, with superior plasticity to adapt to a joint environment, resulting in improved tissue regeneration and repair [8,9,10]. 

NC isolation is usually achieved through collagenase digestion; this step may play an important role in the overall success of the procedure, affecting both the yield and viability of isolated cells. In fact, collagenase treatment which is too intensive may often result in chondrocyte isolation failure [11]; conversely, collagenase treatment which is too mild may provide inadequate cell yield. In this respect, it is not to be excluded that the isolation outcome may be also affected by donor tissue composition, presumably because of individual variations resulting in unique collagen patterns in each donor, as observed in the case of pancreas tissue [12]. Therefore, in prospective clinical uses, tailored digestion protocols might be required for optimal cells yields. 

Current procedures to isolate cells from tissues utilize *Clostridium histolyticum* collagenases to achieve digestion of the extracellular matrix, in order to release chondrocytes. Commercially available collagenases are either recombinant, e.g., ColG and ColH, or a blend of crude collagenase extracted from *C. histolyticum* cultures (containing different percentages of the aforementioned collagenases plus other proteolytic enzymes). Both collagenases contain collagen-binding domains which are able to interact with soluble tropocollagen and insoluble collagen structures, which is essential in dismantling the collagen structure and for subsequent hydrolysis [13,14,15,16]. However, the specific contribution of these collagenases in cartilage digestion is unknown. Moreover, for various commercial blend preparations, information about the specific activity related to each class of collagenases contained therein is not provided [17]. 

This study assesses the relative contribution of each collagenase in cartilage digestion, and seeks to establish an optimized protocol for NC isolation from cartilage using recombinant collagenases, namely ColG and ColH [18,19,20,21,22,23], in order to obtain highly viable cells which will be useful in producing large amounts of ECM component.

## 2. Materials and Methods

### 2.1. Determination of the Enzymatic Activity of Recombinant ColG and ColH 

The recombinant collagenases ColG (145 kDa) and ColH (149 kDa) were produced and chromatographically purified as previously described [23]. 

Enzymatic activity was quantified using the ninhydrin-based assay Collagenase Substrate Kit (Sigma-Aldrich, Milan) according to manufacturer‘s instructions and in the presence of carbobenzoxy-Gly-Pro-Gly-Gly-Pro-Ala-OH synthetic peptide, with some modification [24,25]. In particular, the synthetic substrate was prepared at a concentration of 2.4 mg/mL in 50mM TES Buffer containing 0.36 mM CaCl_2_. The pH was adjusted to 7, instead of 6.3, in order to increase the sensitivity of the assay.

### 2.2. Digestion of Collagen and Cartilage Tissue

Collagen type-I (BD Biosciences, San Jose, CA, USA) and type-II (Advance Biomatrix, Carlsbad, CA, USA) were neutralized by phosphate buffer. Then, 100 μg of each collagen was incubated in a thermomixer with 1 μg (0.0025 U) of ColG or 1 μg (0.03 U) ColH at 37 °C for 15–60 min. After incubation, the protein patterns were analyzed by 7.5% SDS–PAGE assay. 

To evaluate the ability of ColG and ColH to digest the cartilage, the collected tissue was rinsed in medium without serum and then weighed, and divided into aliquots of 1 g per tube. Each aliquot was digested in 10 mL volume with ColG at 1 mg/mL (2.5 U) and ColH 1 mg/mL (30 U) containing Thermolysin (TML) 25 µg/mL (Promega, Milan, Italy) for different digestion times at 37 °C. After digestion, the tissue was processed and filtered with 20-μm nylon filter membrane. The undigested tissue was weighed in order to measure the dissociation percentage. All experiments were carried out in triplicate. Different lots of ColG and ColH were used, having 2.5 and 30 U/mg enzymatic activity, respectively. 

Moreover, in order to analyze the tissue matrix composition before and after digestion, the nasal cartilage samples were minced and mechanically powdered in the presence of liquid nitrogen. Aliquots of 100 mg of powdered tissue were digested for 24 h at 37 °C, as reported in Table 1. The digested and undigested tissue were then loaded onto an SDS PAGE in order to analyze the protein pattern in each condition.

### 2.3. Isolation of Nasal Septal Chondrocytes from Bovine Tissue and Cell Culture Monolayer Expansion

Bovine nasal septa from a 1-year-old animal were obtained from a local abattoir within 12 h of slaughter. Biopsies were harvested and washed with PBS to remove all blood components. One gram of minced tissue was incubated with a mixture of 300 units of ColH plus TML 250 µg in 10 mL of Dulbecco’s modified Eagle’s medium (Sigma, Milan, Italy) containing 1% Pen-Strep and 1% Fungizone (Euroclone, Milan, Italy).

The sample was digested for 18 h at 37 °C in 5% CO_2_. After digestion, the collagenase/chips solution was filtered through a 20-μm nylon filter membrane.

After centrifugation for 10 min at 250 g, the pellet containing chondrocytes was washed twice in DMEM. The cells were than suspended by adding 20 mL of medium, and cell viability was assessed using Trypan Blue exclusion test. Cells were seeded at an initial density of 5 × 10^3^ cells/cm^2^ in T-175 flasks in DMEM supplemented with 10% fetal bovine serum (FBS Sigma, Milan, Italy) + 1% Pen-Strep +1% Fungizone and 50 µg/mL acid ascorbic (Sigma, Milan, Italy). Cell cultures were expanded in an incubator humidified at atmospheric pressure at 37 °C and 5% CO_2_. For viability, assay cells were seeded on 24-well plates at a concentration of 20,000 cells per well, in triplicate. Growth was quantified using Alamar Blue colorimetric assay (Thermo Scientific, USA) according to the manufacturer’s recommendations. Fluorescence was measured with a plate reader using excitation/emission wavelengths of 530/590 nm. The standard curve was obtained using different chondrocyte concentrations.

For 3D culture, chondrocytes were cultured inside 3D collagen gel (Rat collagen I) (BD Biosciences, San Jose, CA, USA). The collagen solution was prepared in Hank’s buffer (2.5 mg/mL) and neutralized with NaOH to induce polymerization of collagen. Immediately afterwards, the cell pellet was resuspended into the neutralized solution. Cells were seeded on 24-well plates and incubated at 37 °C with 5% CO_2_. After polymerization, 1.5 mL of the complete medium was added.

### 2.4. SDS Electrophoresis

Sodium dodecyl sulphate-polyacrylamide gel electrophoresis (SDS-PAGE) was carried out as described by Laemmli et al. [26]. After electrophoresis, the proteins were colored with 0.25% Coomassie Brilliant Blue G-250 (Sigma, Milan, Italy). 

The molecular weight of the enzyme was estimated using a molecular weight marker (Sigma, Milan, Italy).

### 2.5. RNA Isolation and cDNA Synthesis

Total RNA was extracted using Trizol reagent (Invitrogen, CA, USA) according to the manufacturer’s recommendations from chondrocytes cultured in a 2D system for 1 and 10 days, or in 3D collagen type-I hydrogel cultured for 10 days, as well as from nasal bovine cartilage which was previously flash frozen in liquid nitrogen and ground to fine powder using a tissue disruptor.

RNA concentrations and quality were verified by spectrophotometry (optical density (OD) at 260 nm), whereas the RNA integrity was checked using a 1.5 % agarose gel. The RNA was stored at −80 °C for future use. 

The extracted RNA (500 ng) was treated with RNA qualified 1 (RQ1) RNase-Free DNase (Promega, Madison, WI, USA) to remove any residual genomic DNA contamination, and the DNase was inactivated by adding 25 mM EDTA. 

First-strand cDNA was synthesized from 250 ng DNase-treated total RNA samples using random primers and High Capacity cDNA Reverse Transcription Kit (Life Technologies Corporation, Carlsbad, CA, USA), following the manufacturer’s instructions. The cDNA mixture was stored at −20 °C.

### 2.6. RT-qPCR

RT-qPCR was performed using the BIO-RAD CFX96 System with Power Sybr Green as the chemical detection method (Applied Biosystems, Forster City, CA, USA). Real-time PCRs were carried out in 96-well plates in a 20 µL mixture containing 1 µL of a 1:20 dilution of the cDNA preparations, using the following PCR parameters: 95 °C for 10 min, followed by 40 cycles of 95 °C for 10 s, and 60 °C for 60 s. The sequences of the specific primer pairs used for qPCR are shown in Table 2. Samples were run in triplicate. The absence of nonspecific products was confirmed by both the analysis of the melt curves and electrophoresis in 2 % agarose gels. The 18S rRNA, actin β and GAPDH were chosen as reference genes. A normalization factor was calculated based on geometric averaging of the expression level of these reference genes, and was used to quantify the expression levels of the target genes. Quantitative real-time PCR was conducted according to the manufacturer’s recommendations. All data represented relative mRNA expressed as the mean ± S.D. (n = 3). Significant differences between the values of the different treated groups and the reference control groups were determined by one-way ANOVA using Statistica 6.0 (StatSoft, Tulsa, OK, USA).

## 3. Results and Discussions

### 3.1. Cartilage Digestion Using Recombinant Collagenases G and H

Reports of comprehensive experiments aiming to investigate the ability of different collagenase classes to digest extracellular matrix from different tissues are still lacking.

Such information may be useful in optimizing blends of digestion enzymes and, in turn, the overall NCs isolation procedure. While the precise assessment of the specific activity of each enzyme in commercial blends is unreliable, as they are copurified from bacterial cultures, recombinant enzymes offer this unique opportunity. Because they are produced separately, their specific activity can be individually and precisely measured. Therefore, using the recombinant ColG and ColH, with well-defined enzymatic activity, offers the unique possibility of both assessing the specific contribution of each enzyme in tissue digestion and blending them in the most effective ratio, which is expected to result in much higher effectiveness and reproducibility of the isolation protocols.

**Figure 1:**
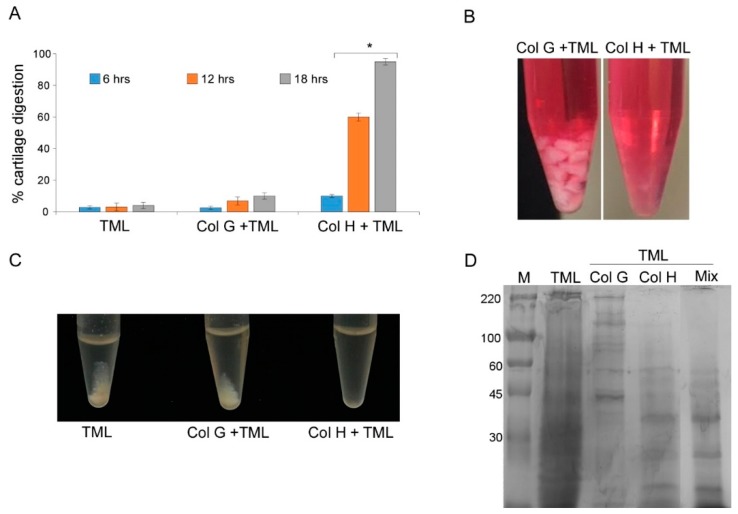
Performance of ColG and ColH in tissue disgregation. (**A**) Cartilage dissociation assay using ColG and ColH in the presence of TML at different times (6, 12, and 18 h). (**B**) Cartilage digested with Col H or G plus TML after 18 h of treatment. (**C**) Samples containing powered cartilage treated 24 h with only TML, ColG plus TML, or ColH plus TML. (**D**) SDS PAGE of protein extracted from cartilage digested with thermolysin (TLM) alone or added of ColG, ColH, or both ColG and H (Mix). M = High molecular weight marker. The data represent the mean ± SD of three independent experiments.

Therefore, we tested such recombinant enzymes in cartilage tissue digestion and compared different protocols for cartilage dissociation. The defined amount (expressed as enzymatic activity) of ColG and ColH per gram of cartilage was used, and the tissue was allowed to dissociate for up to 18 h (Figure 1A). Reactions were carried out also in the presence of a low amount of thermolysin (TML). In addition, cartilage was also incubated with TML alone, at the same concentration, which is known to be unable to digest collagen fibrils. A high rate of cartilage disaggregation was achieved in the presence of ColH plus TML for 18 h at 37 °C, whereas in the same conditions, ColG was ineffective, similarly to what occurred in the presence of TML alone (Figure 1A).

As shown in Figure 1B, after digestion with ColH and TML, the tissue became translucent, a well-known indicator of collagen digestion, which was further confirmed by the efficient release of cells into the medium. In contrast, digestion using ColG and TML failed to efficiently degrade the matrix, thus preventing the release of cells.

In order to obtain further insights into the individual contribution of ColG and ColH in tissue dissociation, powdered tissue was separately digested with same amounts of ColG and ColH (in the presence of TML). The results confirmed that only the mix containing ColH resulted in a very efficient digestion process, and that the presence of ColG did not result in further improvement (Figure 1C).

Total protein patterns resulting from the digestion of powdered cartilage were compared (Figure 1D). Digestion performed with ColG did not result in an evident substrate digestion compared to the total proteins extracted from the cartilage (as reported in materials and methods section); conversely, the treatment with ColH resulted in a more complex pattern of low molecular weight components, where the shift toward a molecular weights below 60KDa and lower fragments (Figure 1D) indicated that a much more efficient collagen digestion had occurred. Moreover, the treatment with the blend of collagenases (ColG + ColH) failed to show further improvements in the pattern modification compared with ColH alone. Altogether, these observations strongly suggest that the ColG activity, if any, is negligible in the digestion of the complex ECM of cartilage.

**Figure 2:**
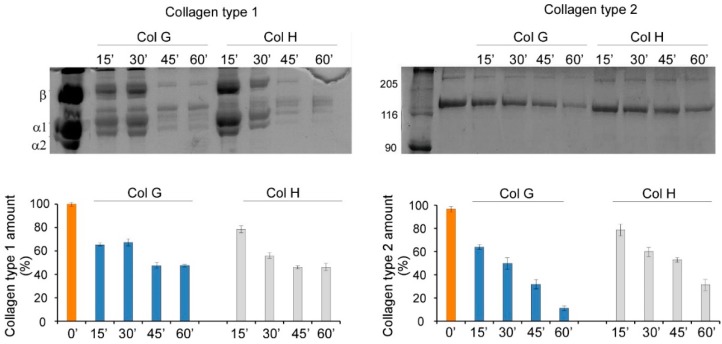
SDS PAGE and densitometric analyses of collagen type I (left panel) and type II (right panel) digested by ColG and ColH. Digestion was performed at different sampling times, i.e., 0’, 15’, 30’, 45’, and 60’.

In order to exclude the possibility that the different digestion performances observed with the two collagenases were due to the low enzymatic activity of the employed ColG preparations toward natural substrates, specific assays were carried out using purified collagens. Thus, both enzymes were incubated with soluble collagen types I and II, and the capability of recombinant ColG and ColH in digesting type-I collagen fibrils over time (from 15 to 60 min) was investigated and assessed by densitometric analyses after gel electrophoresis; similar behavior was observed in the digestion of type-II collagen (Figure 2). 

As expected, densitometric analyses of digested collagens showed relevant activity of both enzymes. In particular, comparable results were obtained with both enzymes in the digestion of type-I collagen. Meanwhile, ColG showed much higher activity toward type-II collagen, compared with ColH.

Therefore, it was confirmed that ColG is fully active, even on purified natural substrates. Its negligible activity in cartilage digestion might be attributable to the possible inhibitory effects of tissue components, which might impair substrate accessibility and/or the overall enzyme activity [27].

### 3.2. Isolation of Nasal Septal Chondrocytes and their characterization

**Figure 3:**
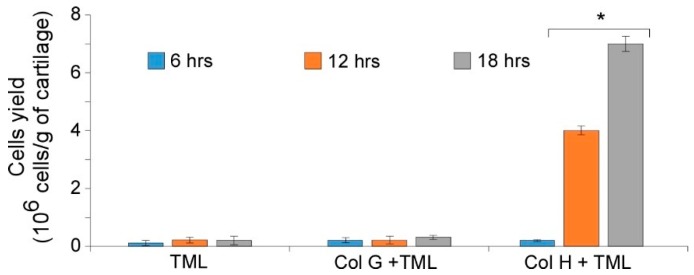
Performance of ColG and ColH in cell release from cartilage using ColG and ColH in the presence of TML at different times (6, 12, and 18 h).

Taking advantage of the previous results, bovine nasal cartilage was allowed to dissociate in the presence of Col G-TML, ColH-TML enzymes blend, or TML alone for further isolation of nasal chondrocytes. Col H was shown to be fully suitable for the digestion of the native cartilage, which accomplished the release of the cells into the medium, as assessed by the quantification of the cells obtained using the different enzyme blend reported in Figure 3. Conversely, no significant recovery of the cells was achieved after cartilage dissociation in the presence of TLM or ColG+TML.

The procedure allowed us to recover ~6–7 × 10^6^ chondrocytes/gram of tissue, with 95 % viability. This result represents a significant improvement in cell yield compared to the only other procedure reported for bovine NC isolation, in which 1.5 × 10^6^ chondrocytes/gram of tissue were obtained [7]. 

In order to further analyze the suitability of isolated chondrocytes for in vitro cells expansion, which is a mandatory prerequisite for any subsequent procedure and application, isolated NCs were put in properly selected culture media. 

Various culture systems have been described for chondrocyte in vitro expansion [28], including 2D and 3D culture conditions, where such cells have shown different propensities to expand and differentiate. In particular, it has been reported that the collagen hydrogel tridimensional system better supports chondrocyte differentiation [29]. Our experiments aimed first to assess the actual expansion potential of the newly-extracted NCs, being the best culture conditions that support differentiation and/or the maintenance of the differentiated state to be more precisely tailored in each case, even from the perspective of specific uses of such cells. Nevertheless, differentiation markers were assessed as well in order to get basic information about the suitability of the employed culture conditions regarding chondrocyte differentiation. Therefore, we set up 2D and 3D collagen type-I hydrogel culture systems in order to assess their performance in supporting chondrocytes expansion.

**Figure 4:**
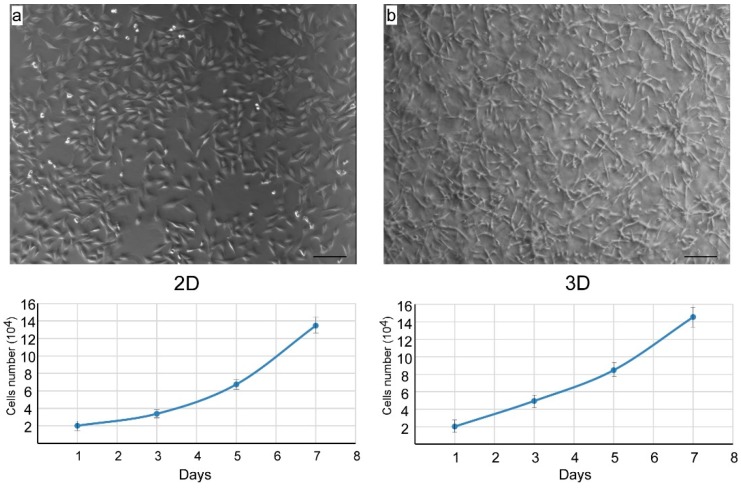
Phase contrast images and growth kinetic curves of chondrocyte isolated with col H plus TML and cultured in 2D (**a**) and 3D (**b**) conditions.

As shown in Figure 4, cells were able to proliferate in both 2D and 3D culture conditions exhibiting uniform morphology with identical size and shape. However, growth in 2D resulted in a better spread of the cells on the substrate, while cells appeared to be distributed on different planes and exhibited a more tapered phenotype when grown in 3D type-I collagen gel.

**Figure 5:**
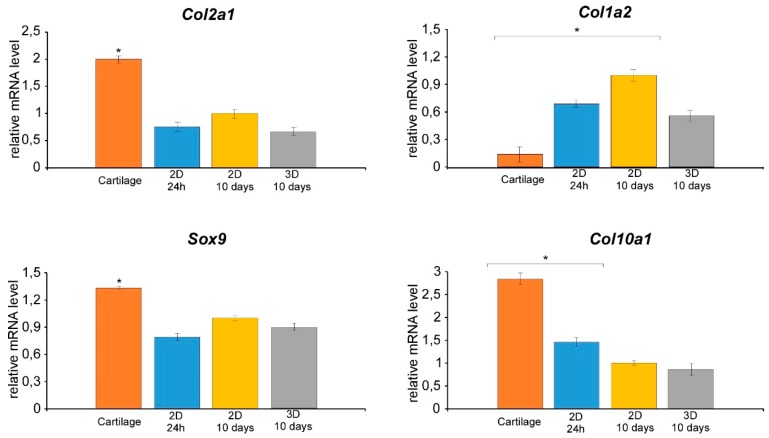
Gene expression analyses of Sox9, Col II, I, and 10 by qRT-PCR. The gene expression levels were analyzed using the 2^−ΔΔCt^ method using β-actin, 18S rRNA, and GAPDH as the internal controls. The data represent the mean ± SD of three independent culture experiments. Bars with different letters are significantly different from each other at *P* < 0.05.

Moreover, in order to monitor the NCs differentiated state after culturing in different conditions, the gene expression of some cartilage-specific phenotypes, including Sox9,Col II, and Col 10a1 [30,31,32,33], as well as Col I as a proxy of fibrous differentiation [34,35,36], was evaluated and compared with that observed in tissue. In particular, gene expression analyses were carried out on primary chondrocytes (24 h after tissue dissociation) and after culturing for 10 days in 2D or in 3D collagen type-I hydrogel. Additionally, gene expression analyses were performed on RNA purified from bovine nasal cartilage, which represents the physiological condition of in vivo differentiation (Figure 5). 

As expected, SOX9 and COL II were overexpressed in cartilage tissue, where the COL10a1 mRNA level, a well-known marker of terminally differentiating chondrocytes, increased. Meanwhile, the gene expression of a fibrous marker, namely, collagen 1, was dramatically reduced. Newly-extracted (24 h cultured), 2D or 3D cultured cells showed a different pattern of gene expression, with variable transcription levels for Sox9 and Col II. Not surprisingly, 2D cultured cells possess some propensity to express Col I mRNA, which has been related to the loss of the chondrocyte phenotype after growth in monolayer [35,36]. However, the Col I transcript was found also in intact and newly isolated articular cartilage [37]. This suggests the possibility of the isolated NCs undergoing different dedifferentiation/differentiation fates (fibroblast vs. chondrogenic phenotype), which mainly rely on the use of specific culture conditions and which, in turn, may lead to the activation of different genes expression patterns. However, these effects are likely related to the culture conditions, rather than to the isolation procedure. Unsurprisingly, Col10a1 mRNA expression in 2D or 3D cultured cells was significantly reduced compared to adult cartilage tissue (Figure 4). However, the COL10a1 mRNA level was slightly higher in primary chondrocytes (24 h of cultivation) than in 10-day, 2D or 3D cultivated cells. Such a result may be explained by the limited duplication steps after 24 h of propagation of primary chondrocytes which maintained some of the characteristics of the original tissue.

## 4. Conclusions

The isolation of a large number chondrocytes from cartilage is a necessary and critical step for tissue repair/regeneration through ACT. Adult cartilage tissue is composed of an abundant family of ECM proteins, among which collagens are the most representative, making their digestion an essential step for the proper purification of cells.

Since collagenase treatment which is too intensive may result in decreased chondrocyte viability, we explored the specific contribution of two major recombinants of *C. histolyticum*, i.e., ColG and H. We showed, for the first time, that the ColH alone (blended with TML) is able to digest the ECM of NCs and to release them from the tissue; conversely, ColG showed a negligible contribution in cartilage digestion, making its presence unnecessary. Similar results were obtained on the carbobenzoxy-Gly-Pro-Gly-Gly-Pro-Ala-OH synthetic peptide, while insignificant differences were found on water-soluble collagens types I and II.

The content and localization of collagen subtypes in different tissues, however, may affect cell recovery.

The procedure herein reported showed high chondrocytes yields, with good proliferative capability in both 2D and 3D cultures, with different propensity to dedifferentiate, as assessed by gene expression analyses. Therefore, it may be worth considering the use of advanced cell culture systems in order to further improve chondrocyte expansion without dedifferentiation.

## Figures and Tables

**Table 1:** Conditions herein used to digest cartilage.

	Exp1	Exp2	Exp3
**ColG**	2.5 U/mL	-	2.5 U/mL
**ColH**	-	30 U/mL	30 U/mL
**TML**	25 µg/mL	25 µg/mL	25 µg/mL

**Table 2:** Oligonucleotide primers used in this study.

Primers	Sequences (5′–3′)	Accession Number
GAPDH	ATCTCGCTCCTGGAAGATG ^a^ TCGGAGTGAACGGATTCG ^b^	NM_001034034
Actin β	TGGGCATGGAATCCTG ^a^ GGCGCGATGATCTTGAT ^b^	NM_173979
18S	TTCGATGGTAGTCGCTGTGC ^a^ TTGGATGTGGTAGCCGTTTC ^b^	NR 036642
Col1A2	GGATGGTCACCCTGGAAAAC ^a^ CCCCTAATGCCCTTGAAGC ^b^	NM_174520
Col2A1	TGATCGTGGTGACAAAGGTG ^a^ ATCTGGGCAGCAAAGTTTCC ^b^	NM_001001135
Sox9	ACGCAGATTCCCAAGACAC ^a^ GGTTTCCAGTCCAGTTTCG ^b^	XM_014478986
Col10A1	CTGGAGTGGGGAAAAGAGG ^a^ TGCCTTCTGGTCCTTGTTC ^b^	NM_174634

^a^ Forward primer, ^b^ Reverse primer.
